# Associations between childhood experiences of parental corporal punishment and neglectful parenting and undergraduate students’ endorsement of corporal punishment as an acceptable parenting strategy

**DOI:** 10.1371/journal.pone.0206243

**Published:** 2018-10-26

**Authors:** Naomi Kitano, Kouichi Yoshimasu, Beverley Anne Yamamoto, Yasuhide Nakamura

**Affiliations:** 1 Research Center for Community Medicine, Wakayama Medical University, Wakayama, Japan; 2 Department of Public Health, School of Medicine, Wakayama Medical University, Wakayama, Japan; 3 Graduate School of Human Sciences, Osaka University, Suita, Japan; 4 Department of Hygiene, School of Medicine, Wakayama Medical University, Wakayama, Japan; 5 Faculty of Nursing and Rehabilitation, Konan Women’s University, Kobe, Japan; Montana State University, UNITED STATES

## Abstract

This study evaluated the effects of childhood experiences of parental corporal punishment (CP) and neglectful parenting (NP) on Japanese university students’ endorsement of parental CP (EPP) to discipline children, in relation to subjective happiness (SH). A total of 536 undergraduate students who showed no physical symptoms completed anonymous paper-based questionnaires addressing demographic characteristics, undergraduate classes, and recent health conditions on SF-8 (PCS, MCS). It was found that the proportions of participants who experienced pervasive CP and NP were larger in men than in women (36.5% vs. 19.4% for CP; 22.1% vs. 9.7% for NP). Multiple regression analyses (n = 346) revealed that the CP score was associated with positive EPP (β = 0.310, p < 0.001). Further, students whose major was nursery education reported significantly lower level of EPP; however, neither SH nor good recent health conditions significantly reduced EPP. The NP score was inversely associated with the SH score (β = -0.253, p < 0.001) (n = 346). In conclusion, childhood experiences of parental CP may affect adolescents’ views related to their own parenting. Further investigation using internationally comparable methodologies, especially in prospective cohort studies, is warranted, not only in Japan but also in other Asian countries.

## Introduction

The prevention of child maltreatment is one of the most urgent public health issues worldwide [1,2]. Recent neurobiological, morphological, genetic, and epigenetic findings have offered experimental evidence to support the relationship between child maltreatment and increased susceptibility to health impairments later in life [3,4]. Furthermore, many longitudinal cohort studies have suggested that child maltreatment and adverse familial environments have negative effects on long-term health [5–8]. Epidemiological studies have consistently shown a high prevalence of mental health problems among children and adolescents in community populations [9]. The heterogeneity in individual responses to such experiences of maltreatment and emotional abuse is an important predictor of poor mental health throughout life [10,11], and this might lead to the perpetuation of child abuse or neglect between generations. Such intergenerational transmission of maltreatment has been reported based on several theoretical models in Western countries [12–14]. The moderating effects of cultural factors on the link between parenting and child outcomes have been inconsistent [15–17]. Across cultural groups, more frequent use of corporal punishment is associated with greater externalizing behaviors in children [18]. However, previous multicultural studies did not include Japan, where parenting styles and discipline tactics are somewhat different compared with even other East Asian countries [19], suggesting that Japan has set comparatively high value on ‘loyalty’ while Korea has set higher value on ‘piety for their parents’. In Japan, a dramatic increase in the number of reported cases of child maltreatment [20] has raised concerns about not only the health and welfare of the children most at risk but also the long-term impacts of corporal punishment (CP) [21] and neglectful parenting (NP). Therefore, we sought to identify young people who experienced childhood parenting problems in a population of university students without physical symptoms to explore the effects of such experiences on young adulthood.

Hence, this study assessed the frequencies of reported parental CP and/or NP and their effects on young adults, focusing on their endorsement of CP as an acceptable parenting strategy and self-reported well-being, such as subjective happiness (SH). We also examined the confounding effects of SH and subjective mental and physical health conditions on the association between experiences of CP and/or NP and endorsement of CP. Those with poor SH or with bad mental health and experiences of CP and/or NP may endorse CP. However, considering the traditional background of home discipline, even those with good SH might strongly endorse CP.

We also hypothesized that students who experienced childhood CP and/or NP would report lower levels of subjective well-being regardless of their most recent physical/mental health status. A previous study has suggested that adolescents’ exposure to maternal negative affective behavior is associated with adolescents’ subjective daily well-being [22]. Happiness is a composite of life satisfaction, coping resources, and positive emotions [23]. Positive emotions are a powerful source of growth and change, predicting both individuals’ judgments about life and their skills for living well, including recovery from illness [24,25].

Additionally, studying nursery education may be a possible factor for the prevention of maltreatment, which is inversely associated with endorsing CP. Children exposed to school-based programs show improvements in protective behaviors and knowledge as well as increase disclosure of abuse [26]. A nursery educational course includes a variety of social sciences such as psychology, psychiatry, and welfare, including prevention or coping with child maltreatment.

As such, the main purpose of this study was to examine the association between childhood experiences of parental CP and/or NP and endorsement of CP in relation to current subjective well-being in apparently healthy Japanese university students. Next, to explore whether the endorsement of CP can be changed by students’ appropriate recognition, we also examined whether university students who study nursery education are less likely to endorse CP as a suitable parenting practice.

## Material and methods

The present study was an exploratory, cross-sectional investigation targeting undergraduate students at five universities in Western Japan. It was conducted from September 2007 to July 2008. The study protocol was reviewed and approved by the Research Ethics Committee of the Graduate School of Human Sciences, Osaka University. Completing and returning the anonymous questionnaires indicated participants’ tacit informed consent.

### Study participants

Participants were recruited from first- and second-year undergraduate classes in nursery education, health nursing, social welfare, domestic science, nutrition, and psychosociology. Eligible students were those studying in the Kansai area who could be contacted by the first author (NK), who was the principal investigator, in cooperation with co-investigators at multiple universities. A total of 692 students from five universities were recruited. Valid responses were received from 536 respondents (104 men and 432 women, valid response rate: 77.5%). None of the study participants had any experience in caring for children.

### Definitions

In the present study, the definition of CP reflected the definitions by the American Association of Pediatrics [27] and Straus and Donnelly [28]. We defined CP as the use of physical force, including spanking and hitting, for correcting or controlling a child’s behavior.

We employed the definition of NP behaviors developed by Straus and Kantor [29]. NP behaviors are those that constitute a failure to act in ways presumed by a society’s culture to be necessary to meet a child’s developmental needs and that are the caregiver’s responsibility to provide.

### Measurements

We constructed a five-part questionnaire, drawing on existing instruments for use in the general population. Our self-reported anonymous questionnaire (paper-based) covered demographic characteristics, experiences of CP and NP, recent physical and mental health conditions, endorsement of parental use of CP, and subjective well-being. Since no official Japanese version existed for a pre-existing instrument, we conducted a translation using cross-cultural translation methodology and validation based on Acquadro et al. [30], and we checked the reliability of these translations by confirmatory factor analyses and calculation of Cronbach’s α coefficients.

### Demographic data

The first part of the questionnaire collected basic demographic data (i.e., gender, age, number of siblings, birth-order, mother’s and father’s ages, and years of education completed by parents) and basic information about the participant’s academic affiliation and major (i.e., university classes).

### Experience of parental corporal punishment

To assess participants’ experience of parental CP, two items were translated into Japanese and administered: one asked whether respondents had been hit or spanked “a lot” before the age of 12, and the other asked whether they were hit “a lot” during their teenage years [31]. These items were chosen because they have been used internationally [31], facilitating comparisons between findings.

Any response to these two items other than “strongly disagree” was highly suggestive of the respondent having experienced CP because of the inclusion of the word “a lot” [31]; even if they disagreed with “a lot,” they might agree with “a little.” Thus, for either question, responses of “strongly agree,” “agree,” and “disagree” were considered suggestive of CP, and responses of “agree” and “strongly agree” were considered to indicate pervasive CP.

To form the predictor measure in this study, responses to the two items concerning CP (strongly agree = 4, agree = 3, disagree = 2, and strongly disagree = 1) were summed to produce the score representing the experience of CP (CP score).

### Experience of neglectful parenting

To assess participants’ experience of NP, the eight-item revised version of the Multidimensional Neglectful Behavior Scale Adult Recall Short Form (revised MNBS-AS) was employed [32]: two items concerning physical neglect (e.g., my parents did not keep me clean), two items concerning cognitive neglect (e.g., my parents did not help me to do my best), two items concerning emotional neglect (e.g., my parents did not comfort me when I was upset), and two items concerning supervisory neglect (e.g., my parents did not care if I did things like shoplifting). The revised MNBS-AS was developed as a brief instrument for use in surveys targeting general populations [29,32,33].

As a predictor measure in the study, responses to the eight items of the revised MNBS-AS were rated (strongly agree = 4, agree = 3, disagree = 2, and strongly disagree = 1) and summed to produce the score representing the experience of NP (NP score). To distinguish between single and multiple experiences of NP, we followed Straus and Savage [33] by presenting separate data about the percentage of students who reported three or more experiences of NP, which was evidence of “pervasive NP.”

### Recent health condition

This part of the questionnaire concerned respondents’ self-reported physical and mental health condition over the past month. The official Japanese version of the 8-item SF-8 Health Survey was administered (e.g., During the past four weeks, how much did physical health problems limit your physical activities [such as walking or climbing stairs]? and During the past four weeks, how much have you been bothered by emotional problems [such as feeling anxious, depressed, or irritable]?) [34]. Two indicators, the physical condition score (PCS) and mental condition score (MCS), were calculated as indicators of respondents’ recent health condition.

### Subjective happiness

To capture each participant’s subjective well-being, we administered the official Japanese version of the Subjective Happiness Scale, a global SH seven-point Likert scale consisting of four items (e.g., Compared with most of my peers, I consider myself: less happy (1–7) more happy; Some people are generally very happy. They enjoy life regardless of what is going on, getting the most out of everything. To what extent does this characterization describe you?) [35,36]. In the present study, the SH score—the mean score of the four items, assessed on the seven-point Likert scale—was used as an outcome measure of subjective well-being. The degree of SH rises as the SH score increases.

### Endorsement of parental use of physical punishment on children as a form of discipline

The Adult Adolescent Parenting Inventory-2 (AAPI-2) was developed to assess the parenting and child-rearing attitudes of adults and adolescents, regardless of experience in raising their own children [37]. We obtained permission to translate this instrument into Japanese, and its translated contents were confirmed using back-translation. In the present study, our Japanese version of AAPI-2 Form B was administered. We modified the original five-point Likert scale to a four-point scale (strongly disagree, disagree, agree, and strongly agree) in this study to avoid Japanese people’s tendency to choose the “uncertain” response [38].

Following the 2005 edition of the *AAPI Online Development Handbook* [37], we performed a confirmatory factor analysis for each subscale of AAPI-2 Form B in our sample. We identified one factor (Cronbach’s α = 0.769) consisting of six items regarding the endorsement of the use of physical punishment (e.g., “Spanking children when they misbehave teaches them how to behave”). We called this factor the “Endorsement of Physical Punishment as a Form of Discipline Indicator” (EPP). The response to each EPP item was rated as follows: strongly agree = 4, agree = 3, disagree = 2, and strongly disagree = 1. Adding the response ratings from the six EPP items produces the EPP score, which was used as an outcome measure in this study. The degree of endorsement of physical punishment rises as the EPP score increases.

### Statistical methods

First, descriptive analyses were performed to examine respondents’ demographic characteristics and the frequency distributions of responses to items concerning experience of CP and NP. To examine the differences between the two groups of responses, *t*-tests and chi-square tests or Fisher’s exact test were conducted for parametric and non-parametric variables, respectively.

Next, using data of 346 participants (56 men and 290 women), we conducted multiple regression analyses to detect the independent contributions of the predictors of CP and NP scores to the EPP score as outcome variable, adjusting for participants’ basic demographic factors, undergraduate class, recent health condition on SF-8 (PCS, MCS), and with/without the SH score. As preparation for the multivariate analysis, bivariate correlation analyses were performed to examine the associations among the independent variables, using list-wise case deletion. Variance inflation factors (VIFs) were also used as diagnostic for multicollinearity. Another multiple regression analysis was conducted using the SH score as an outcome variable to examine the independent effects of CP and NP scores on the SH score.

For all statistical analyses, SPSS version 24.0 for Windows (IBM Corp., Armonk, NY, USA) were used. A two-tailed *P*-value of less than 0.05 was required for statistical significance.

## Results

As shown in Table 1, there were no statistically significant gender differences in the sociodemographic characteristics or present status of the 536 respondents, except for the variable “has studied health or welfare at university.”

**Table 1:** Characteristics of the study participants (n = 536).

	Male (n = 104) Mean (SD) / n (%)	Female (n = 432) Mean (SD) / n (%)	*P*-value*
**Socio-demographic variables**			
Age	19.4 (0.71)	19.5 (0.96)	0.693
Number of siblings^†^	2.5 (0.75)	2.5 (0.73)	0.785
Birth-order^‡^	1.7 (0.79)	1.8 (0.79)	0.670
Mother’s age at birth^§^	28.1 (4.1)	28.1 (3.9)	0.970
Age difference between mother and father (years) ^ǁ^	-2.9 (4.2)	-2.7 (3.5)	0.607
Mother’s years of education^¶^	13.2 (1.6)	13.3 (1.6)	0.746
Father’s years of education^#^	13.8 (2.1)	14.0 (2.0)	0.357
**Undergraduate classes**			
Nursery education	21 (20.2%)	127 (29.4%)	0.059
Health nursing	11 (10.6%)	123 (28.5%)	<0.001
Social welfare	51 (49.0%)	49 (11.3%)	<0.001
Domestic science	0	62 (14.4%)	<0.001
Nutrition	15 (14.4%)	39 (9.0%)	0.101
Psychosociology	6 (5.8%)	32 (7.4%)	0.674
**Recent health condition on SF-8**			
Physical condition score (PCS)	48.9 (7.4)	51.3 (6.4)	0.014
Mental condition score (MCS)	45.9 (8.7)	43.4 (7.3)	0.025
Predictive and outcome measures**			
Score representing experience of corporal punishment (CP score)	4.3 (1.6)	3.4 (1.5)	<0.001
Score representing experience of neglectful parenting (NP score)	15.1 (2.8)	12.8 (3.3)	<0.001
Subjective happiness (SH) score	4.6 (1.1)	4.7 (0.94)	0.836
Endorsement of parental use of physical punishment on children (EPP) score	13.1 (3.4)	12.7 (2.7)	0.413

*Continuous variable: t-test; Categorical variable: chi-square test or Fisher’s exact test

†n = 530 (104 males and 426 females)

‡n = 521 (103 males and 418 females)

§n = 462 (74 males and 388 females)

ǁn = 437 (70 males and 367 females)

¶n = 476 (87 males and 389 females)

#n = 461 (85 males and 376 females)

**n = 346 (56 males and 290 females).

### Experience of parental corporal punishment

Table 2 shows the frequency distribution of the number and percentage of responses to the two items concerning experience of parental CP. Responses of “strongly agree,” “agree,” or “disagree” on either or both of the items were considered suggestive of CP, whereas responses of “agree” or “strongly agree” were considered to indicate pervasive CP.

**Table 2:** Frequency distribution of responses to each question on experience of parental corporal punishment and neglectful parenting in childhood and adolescence (n = 536: 104 men and 432 women).

**Corporal punishment item**		Strongly disagree n (%)	Disagree n (%)	Agree n (%)	Strongly agree n (%)
When I was less than 12 years old, I was spanked or hit a lot by my mother or father.	Men	29 (27.9)	40 (38.5)	29 (27.9)	6 (5.8)
	Women	188 (43.5)	165 (38.2)	57 (13.2)	22 (5.1)
When I was a teenager, I was hit a lot by my mother or father.	Men	40 (38.5)	46 (44.2)	14 (13.5)	4 (3.8)
	Women	245 (56.7)	151 (35.0)	28 (6.5)	8 (1.9)
**Revised MNBS-AS item (sub-category of neglect)**		Strongly disagree	Disagree	Agree	Strongly agree
My parents helped me when I had trouble understanding something.^1^	Men	2 (1.9)	18 (17.3)	63 (60.6)	21 (20.2)
(cognitively)	Women	10 (2.3)	61 (14.1)	247 (57.2)	114 (26.4)
My parents did not comfort me when I was upset.	Men	15 (14.4)	56 (53.8)	27 (26.0)	6 (5.8)
(emotionally)	Women	121 (28.0)	211 (48.8)	80 (18.5)	20 (4.6)
My parents helped me when I had problems.^1^	Men	3 (2.9)	29 (27.9)	52 (50.0)	20 (19.2)
(emotionally)	Women	7 (1.6)	73 (16.9)	230 (53.2)	122 (28.2)
My parents did not care if I did things like shoplifting.	Men	60 (57.7)	30 (28.8)	11 (10.6)	3 (2.9)
(supervisory)	Women	320 (74.1)	104 (24.1)	7 (1.6)	1 (0.2)
My parents did not help me to do my best.	Men	24 (23.1)	56 (53.8)	17 (16.3)	7 (6.7)
(cognitively)	Women	220 (50.9)	188 (43.5)	20 (4.6)	4 (0.9)
My parents gave me enough clothes to keep me warm.^1^	Men	2 (1.9)	8 (7.7)	52 (50.0)	42 (40.4)
(physically)	Women	8 (1.9)	16 (3.7)	147 (34.0)	261 (60.4)
My parents did not keep me clean.	Men	46 (44.2)	47 (45.2)	7 (6.7)	4 (3.8)
(physically)	Women	297 (68.8)	120 (27.8)	9 (2.1)	6 (1.4)
My parents did not care if I got into trouble in school.	Men	51 (49.0)	42 (40.4)	10 (9.6)	1 (1.0)
(supervisory)	Women	284 (65.7)	131 (30.3)	11 (2.5)	6 (1.4)

1: reverse-scored item

Overall, 59.5% of students (72.1% of men and 56.5% of women) provided responses suggestive of experience of CP, indicating that they were spanked or hit before the age of 12 by their parents. Regarding their teenage years, 46.8% of students (61.5% of men and 43.3% of women) gave responses suggestive of experience of parental CP.

The percentage of respondents who gave answers suggestive of experience of pervasive CP was 22.8%. There was a significant gender difference, with 36.5% of men and 19.4% of women (chi-square = 13.932, df = 1, *P* < 0.001) agreeing or strongly agreeing with either or both items concerning experience of CP. There was a strong association between experience of pervasive CP before the age of 12 and during the teenage years (chi-square = 146.504, df = 1, *P* < 0.001).

### Experiences of neglectful parenting behaviors

Table 2 presents the frequency distribution of responses to each of the eight items used to assess experience of NP. Emotional and cognitive forms of NP were reported more frequently than were physical and supervisory forms.

The percentage of respondents who gave answers suggestive of experience of pervasive NP was 12.2%. There was a significant gender difference, with 22.1% of men compared with 9.7% of women (chi-square = 12.081, df = 1, *P* = 0.001) reporting experience of pervasive NP.

### Gender differences in predictor and outcome measures

The mean ± SD of the CP score among 346 respondents (56 men and 290 women) was 3.5 ± 1.6 (range: 2–8, median: 3, skewness: 0.886, kurtosis: 0.196); as shown in Table 1, there was a significant gender difference in the mean CP score (*P* < 0.001).The mean ± SD of the NP score among them was 13.1 ± 3.4 (range: 8–24, median: 13, skewness: 0.427, kurtosis: −0.215); as shown in Table 1, there was a significant gender difference in mean NP scores (*P* < 0.001). The mean ± SD of the SH score among them was 4.6 ± 0.97 (range: 2–7, median: 4.5, skewness: 0.083, kurtosis: −0.151); as shown in Table 1, there was no significant gender difference in mean SH scores (*P* = 0.836).The mean ± SD of the EPP score among 346 respondents (56 men and 290 women) was 12.8 ± 2.8 (range: 6–21, median: 13, skewness: −0.028; kurtosis: 0.269); as shown in Table 1, there was no significant gender difference in mean EPP scores (*P* = 0.413).

### Associations between predictor and outcome variables

As shown in Table 3, a few variables were significantly correlated with EPP or SH scores, but no multicollinearity was detected among the study variables by VIFs. Hence, we included some non-significant variables into the regression models based on the previous evidence. As shown in Table 4, by multiple regression analysis, the CP score had a significantly positive effect on the EPP score, whereas the NP score had no significant effect on the EPP score. The robustness of the standardized partial regression coefficient of the CP score was demonstrated by controlling for respondents’ basic demographic factors, the NP score, undergraduate classes, and PCS and MCS (adjusted R^2^ = 0.135). Subsequently, after the inclusion of the SH score in the model, the results did not change (ΔR^2^ = 0.001) and the CP score had a significantly positive effect on the EPP score (β = 0.310, *P* < 0.001). Another independent variable with significant coefficients was “having studied nursery education,” which showed a significant inverse effect on the EPP score (β = −0.239 in the final model, *P* < 0.001).

**Table 3:** Pearson’s correlation coefficients by bivariate correlation analyses among study variables (list-wise, n = 346).

	①	②	③	④	⑤	⑥	⑦	⑧	⑨	⑩	⑪	⑫	⑬	⑭	⑮	⑯
①	1															
②	0.320***	1														
③	0.122**	0.246***	1													
④	-0.044	-0.210***	-0.254***	1												
⑤	0.018	-0.078*	0.036	0.021	1											
⑥	-0.015	-0.056	0.033	0.042	-0.052	1										
⑦	-0.048	-0.070*	0.019	0.039	0.056	0.512***	1									
⑧	0.022	-0.048	0.042	0.032	0.102**	0.153***	0.500***	1								
⑨	-0.068	0.083*	-0.043	-0.006	-0.020	-0.015	0.019	0.242***	1							
⑩	-0.057	-0.128***	-0.089**	0.021	0.085*	-0.035	0.007	0.153***	0.054	1						
⑪	-0.040	-0.084*	-0.160***	0.034	0.056	-0.134***	-0.075*	0.068	-0.143***	0.412***	1					
⑫	-0.181***	-0.065	-0.067	0.050	-0.138***	0.136***	0.036	-0.008	0.061	-0.065	-0.084*	1				
⑬	-0.017	0.026	-0.024	-0.122**	-0.174***	-0.077*	-0.139***	-0.123**	0.007	0.061	0.068	-0.499***	1			
⑭	-0.071*	-0.032	-0.064	0.132***	-0.013	0.036	-0.005	0.022	0.020	-0.037	-0.027	-0.102**	0.110**	1		
⑮	-0.048	0.020	0.023	-0.121**	-0.067	0.058	0.057	0.012	0.005	0.132***	0.079*	-0.073*	0.136***	-0.186***	1	
⑯	-0.016	-0.027	-0.232***	0.011	0.037	-0.024	0.106**	0.072*	0.009	0.150***	0.055	-0.041	0.029	0.073*	0.269***	1

*** *p* < 0.01

** *p* < 0.05

* *p* < 0.1

①Endorsement of parental use of physical punishment on children (EPP) score, ②Score representing experience of corporal punishment (CP score), ③Score representing experience of neglectful parenting (NP score), ④Gender (Male = 0, Female = 1), ⑤Age, ⑥Number of siblings, ⑦Birth-order, ⑧Mother’s age at birth, ⑨Age difference between mother and father (years), ⑩Mother’s years of education, ⑪Father’s years of education, ⑫Studied nursery education (yes = 1, no = 0), ⑬Studied health nursing/social welfare (yes = 1, no = 0), ⑭Physical condition score on SF-8 (PCS), ⑮Mental condition score on SF-8 (MCS), ⑯Subjective happiness (SH) score

**Table 4:** The results of multiple regression analysis (Dependent variable: Degree of students’ endorsement of physical punishment by parents, EPP score; n = 346).

	Unadjusted β	Adjusted		
Independent variable		*B*	(95%CI)	Β
(constant)		13.904	(6.175, 21.632)	
Score representing experience of corporal punishment (CP score)	0.320***	0.564	(0.373, 0.754)	0.311****
Score representing experience of neglectful parenting (NP score)	0.122	0.012	(-0.078, 0.101)	0.014
Gender	−0.044	0.200	(-0.611, 1.012)	0.026
Age	0.018	−0.051	(-0.360, 0.259)	−0.017
Number of siblings	−0.015	0.276	(-0.210, 0.762)	0.067
Birth-order	−0.048	−0.431	(-0.930, 0.067)	−0.116
Mother’s age at birth	0.022	0.074	(-0.017, 0.165)	0.100
Age difference between mother and father	−0.068	−0.08	(-0.163, 0.003)	−0.102
Mother’s years of education	−0.057	−0.015	(-0.210, 0.181)	−0.008
Father’s years of education	−0.040	−0.053	(-0.208, 0.101)	−0.039
Studied nursery education	−0.181**	−1.574	(-2.370, -0.778)	−0.239****
Studied health and nursing/social welfare	−0.017	−0.686	(-1.388, 0.016)	−0.120
Physical condition score (PCS)	−0.071	−0.039	(-0.083, 0.005)	−0.092
Mental condition score (MCS)	−0.048	−0.024	(-0.063, 0.014)	−0.065

****: *p* < 0.001

***: *p* < 0.01

**: *p* < 0.05

Gender was coded as man = 0 and woman = 1. Studied nursery education and studied health and nursing/social welfare were coded as yes = 1 and no = 0. PCS: Physical condition score on SF-8; MCS: Mental condition score on SF-8

As shown in Table 5, by simple regression analysis, the NP score had a significantly negative effect on the SH score, whereas the CP score had no significant effect on the SH score. The robustness of the standardized partial regression coefficient of NP score was demonstrated by controlling for respondents’ basic demographic factors, the CP score, undergraduate classes, and PCS and MCS (adjusted R^2^ = 0.142). Other independent variables with significantly positive coefficients were MCS, PCS, birth-order, and mother’s years of education.

**Table 5:** The results of multiple regression analysis (Dependent variable: Degree of students’ subjective happiness, SH score; n = 346).

	Unadjusted β	Adjusted		
Independent variables		*B*	(95%CI)	β
(constant)		1.606	(-1.020, 4.232)	
Score representing experience of corporal punishment (CP score)	-0.027	0.029	(-0.036, 0.094)	0.047
Score representing experience of neglectful parenting (NP score)	-0.232****	-0.073	(-0.103, -0.042)	-0.253****
Gender	0.011	-0.079	(-0.355, 0.197)	-0.03
Age	0.037	0.044	(-0.061, 0.149)	0.044
Number of siblings	-0.024	-0.151	(-0.317, 0.014)	-0.108
Birth order	0.106**	0.175	(0.005, 0.344)	0.138**
Mother’s age at birth	0.072	0.003	(-0.028, 0.034)	0.013
Age difference between mother and father	0.009	-0.008	(-0.036, 0.020)	-0.03
Mother’s years of education	0.150***	0.07	(0.004, 0.136)	0.117**
Father’s years of education	0.055	-0.027	(-0.080, 0.025)	-0.059
Studied nursery education	-0.041	-0.025	(-0.295, 0.245)	-0.011
Studied health and nursing/social welfare	0.029	-0.045	(-0.283, 0.194)	-0.023
Physical condition score (PCS)	0.073	0.018	(0.003, 0.033)	0.126**
Mental condition score (MCS)	0.269****	0.037	(0.023, 0.050)	0.286****

****: *p* < 0.001

***: *p* < 0.01

**: *p* < 0.05.

Gender was coded as man = 0 and woman = 1. Studied early nursery education and studied health and nursing/social welfare were coded as yes = 1 and no = 0. PCS: Physical condition score on SF-8; MCS: Mental condition score on SF-8

## Discussion

The present results indicate that a sizable proportion of the undergraduate student participants had experienced pervasive parental CP and NP, and more men than women reported both types of experience (36.5% vs. 19.4% for CP; 22.1% vs. 9.7% for NP). This result is especially interesting given that we targeted an apparently healthy and academically successful population of young people. In a previous Japanese survey [39], it was reported that 21% of junior high school students and 26% of high school students experienced CP from fathers, which is similar with the current findings. To the best of our knowledge, however, the present study shows the first internationally comparable Japanese data on experiences of CP and/or NP targeting young people in a non-clinical setting.

In our study, male respondents were more likely than their female counterparts to have experienced CP and NP as children and teenagers. Externalizing problems were reported to be more critical in boys, while internalizing symptoms were more apparent among girls [40]. Such gender differences may partially explain why males were more likely to have experienced child maltreatment. There is a need to explore the gendered norms and values around childrearing that may influence this outcome. Oshio and Umeda [41] reported that the intergenerational impact of parents’ experience of childhood abuse on problem behavior in children between the ages of 2 and 18 was largely characterized by same-gender linkages: daughters’ problem behavior was more closely associated with childhood abuse of mothers than of fathers, and sons’ problem behavior was more closely associated with their fathers’ experiences. Thus, there is a need to consider gender when developing interventions to address their parenting skills, and attempts to ameliorate the impact of childhood maltreatment may also require gender-sensitive approaches.

CP has been shown to be an ineffective tool for improving child behaviors [27]. Regardless of culture, religion, location, or country, from a human rights perspective, CP of children should be avoided [42,43]. However, despite legal restrictions on the use of CP in school settings, CP is still widely endorsed among the general population. For example, the Japanese General Social Survey 2000/2001 reported that more than half of 5,656 randomly sampled adults approved of the use of CP as a means of discipline, not only by parents but also by school teachers [44]. In addition, the use of CP by Japanese teachers in the context of extracurricular activities has recently emerged as a controversial issue in the media on several occasions. CP’s wide acceptance as a disciplinary tool by the general population may explain the high reported incidence of CP among the respondents in our study—a relatively privileged group of young people. There is a need to create community-wide structures to support positive parenting [43,45].

The revised MNBS-AS has been shown to be reliable and valid in diverse cultural settings (33 universities in 17 countries), including Asian countries such as Hong Kong, Korea, and Singapore. Straus and Savage [33] reported that the proportion of Korean university students with experience of NP was the highest among these three countries. They found that the percentage of those reporting NP was considerably higher for women than for men (41.2% vs. 28.1%). Interestingly, this pattern of gender difference was reversed in our study, with men reporting a higher incidence of NP than women. This difference in findings indicates that, even among culturally close East Asian (Confucian) cultures, there may be considerable diversity in the effects of NP on men and women. These parenting deficits include the use of coercive and punitive parenting strategies that intensify and perpetuate child behavioral problems, thereby increasing the likelihood of further child maltreatment in the family.

The present study revealed the long-term impacts of experience of parental CP and/or NP behaviors. We presented two clear findings. First, undergraduate students who had experienced CP positively endorsed the use of parental CP of children as a form of discipline. This finding is in line with the findings of previous international studies [46,47]. Furthermore, experiencing child maltreatment has been associated with greater use of intimate partner violence later in life [48]. However, in the present study, it should be noted that subjective happiness as well as mental and physical health status were not significantly associated with a reduction in the endorsement of the use of CP on children, which suggests that the endorsement of CP as an acceptable parenting strategy is not affected by subjectively perceived well-being or health conditions.

In terms of preventing intergenerational continuity of tolerant attitudes toward violent behaviors, an important finding of the present study was that respondents who had studied nursery education were significantly less likely to approve of CP of children as a disciplinary tool. Developmental psychology included in the nursery education course may be effective for prevention of CP. This result points to the possibility of educational interventions to sensitize students to problems associated with using CP to discipline children. As per a publication from the World Health Organization Regional Office for Europe [49], “school-based safety education programs aim to teach children to recognize potentially harmful situations and to distinguish between appropriate and inappropriate forms of touching” and “education campaigns, other proactive initiatives can be used to challenge norms that support or justify hitting a child”; however, “the need for school teachers to receive good quality specialist training to deliver these programs should not be underestimated.” There is evidence of improvements in protective behaviors and knowledge among children exposed to school-based programs, regardless of the type of program [50]. The corrective capability of university child-nurturing and educational interventions should be noted.

The second main finding was that the SH score was inversely correlated with the score of experience of parental NP after adjusting for gender, birth order, mother’s and father’s years of education, and both mental and physical health status in the previous month. To the best of our knowledge, in Japan, this is the first study to demonstrate the association between experiences of neglectful behaviors by parents and SH among apparently healthy university students. This result was consistent with those of two previous studies. Milevsky et al. [51], examining the association between parenting styles in adolescence and indices of adolescent psychological well-being, showed that high school students who had experienced neglectful parenting styles scored lower on self-esteem than did students with a parent or parents implementing an authoritative or permissive parenting style. Oshio et al. [52] demonstrated that the experience of childhood adversity had a substantial negative impact on adulthood subjective well-being among Japanese community residents; the participants’ age range was older and wider (25–50 years). Another study in Japan [53] showed that neglect, emotional abuse, and sexual maltreatment predicted borderline personality traits and depression among university students. Although this previous study did not directly evaluate SH, the findings are generally consistent with ours. In addition, children who were left behind by parents who migrated to urban areas were reported to have poorer SH than those who were not left behind in China [54], which is also consistent with our results.

Our study has several limitations. First, it had a cross-sectional design; therefore, we cannot discuss causality between the predictors and outcomes. Additionally, because the respondents of the present study were not recruited randomly, our findings cannot be considered directly generalizable to Japanese young adults, especially for low-education and low-income sectors of the population. Second, the predictor measures were retrospectively collected and based on self-reported data, which are susceptible to respondents’ recall bias and their tendency to choose more socially desirable answers. However, the validity of self-report approaches to studying child abuse and neglect has been examined [55–57], with findings indicating that this approach is valid because official records are likely to underestimate the prevalence of child abuse. Third, students who were exposed to positive forms of parenting and teaching or those who are interested in children may be more likely to study nursery education as well as less likely to endorse CP as an acceptable parenting strategy. In this study, we did not collect information on which our participants were exposed to positive forms of parenting in their childhood, but such participants seem more likely to study an undergraduate course in nursery education. Such factors other than studying nursery education may explain the inverse association between studying nursery education and endorsement of CP.

Moreover, the Japanese-translated version of revised MNBS-AS used in our study has not yet been standardized, since this is the first exploratory study applying MNBS to a Japanese population. To address cross-cultural translation issues in the present questionnaires, at a preliminary research stage, we back-translated the items and checked their reliability according to the methodology recommended by Acquadro et al. [30]. Moreover, we regarded “disagree” responses as suggestive of CP, as mentioned earlier, because participants were asked to report whether they had been hit or spanked “a lot” in the past. However, there might have been differences in interpretation of this phrase among the participants (i.e., some participants might consider “a lot” to mean weekly, whereas others might interpret it to mean daily).

## Conclusion

The high proportion of reports of experience of CP and/or NP in a population of apparently healthy undergraduate students should not be ignored. We clarified that the experience of parental CP in childhood and adolescence was positively associated with undergraduate students’ endorsement of CP as a disciplinary tool to use on children. We also confirmed that experiences of NP were associated with lower levels of SH. However, neither SH nor good mental/physical health conditions significantly reduced endorsement of parental CP to discipline children, indicating that people who endorse parental physical punishment do not always have a mental illness. In addition, at university age, studying nursery education was significantly associated with a reduction in participants’ endorsement of CP; this may suggest the possibility of intervention to stop intergenerational continuity of CP. Future studies could investigate the effects of a nursing education program on beliefs and practices in a longitudinal manner.

In terms of health promotion and the promotion of non-violent forms of parenting, this study presents the possibility of educational interventions that include teaching about child development and effective parenting or nurturing skills. However, the current study was exploratory. Given our results, further investigation using internationally comparable methodologies, especially in prospective cohort studies, is warranted, not only in Japan but also in other Asian countries.
